# Mucosal SARS-CoV-2 S1 adenovirus-based vaccine elicits robust systemic and mucosal immunity and protects against disease in animals

**DOI:** 10.1128/mbio.02170-24

**Published:** 2024-12-04

**Authors:** Najwa D. Aljehani, Levi Tamming, Muhammad Yasir Khan, Rwaa H. Abdulal, Mohamed A. Alfaleh, Aishah Ghazwani, Asalah Helal, Reem M. Alsulaiman, Mohammad A. Sanki, Khalid Alluhaybi, Farah Ayman Sukareh, Rahaf H. Alharbi, Faris H. Alyami, M-Zaki ElAssouli, Salima Shebbo, Wesam H. Abdulaal, Abdullah Algaissi, Ahmad Bakur Mahmoud, Mohammad Basabrain, Diana Duque, Jegarubee Bavananthasivam, Wangxue Chen, Lisheng Wang, Simon Sauve, Turki S. Abujamel, Tarfa Altorki, Rowa Alhabbab, Anh Tran, Xuguang Li, Anwar M. Hashem

**Affiliations:** 1 Vaccines and Immunotherapy Unit, King Fahd Medical Research Center, King Abdulaziz University, Jeddah, Saudi Arabia; 2 Department of Biochemistry, Faculty of Science, King Abdulaziz University, Jeddah, Saudi Arabia; 3 Centre for Oncology, Radiopharmaceuticals and Research Biologics and Radiopharmaceutical Drug Directorate, Health Products and Food Branch (HPFB), Health Canada and WHO Collaborating Center for Standardization and Evaluation of Biologicals, Ottawa, Ontario, Canada; 4 Department of Biochemistry, Microbiology and Immunology, Faculty of Medicine, University of Ottawa, Ottawa, Ontario, Canada; 5 Department of Pharmaceutics, Faculty of Pharmacy, King Abdulaziz University, Jeddah, Saudi Arabia; 6 Department of Pharmacology and Toxicology, Faculty of Pharmacy, King Abdulaziz University, Jeddah, Saudi Arabia; 7 College of Dental Medicine, QU Health, Qatar University, Doha, Qatar; 8 Department of Medical Laboratories Technology, College of Applied Medical Sciences, Jazan University, Jazan, Saudi Arabia; 9 College of Applied Medical Sciences, Taibah University, Almadinah Almunwarah, Saudi Arabia; 10 Department of Medical Laboratory Technology, Faculty of Applied Medical Sciences, King Abdulaziz University, Jeddah, Saudi Arabia; 11 Human Health Therapeutics Research Center, National Research Council Canada, Ottawa, Ontario, Canada; 12 Department of Clinical Microbiology and Immunology, Faculty of Medicine, King Abdulaziz University, Jeddah, Saudi Arabia; The Catholic University of America, Washington, DC, USA; The Catholic University of America, Washington, DC, USA

**Keywords:** vaccine, SARS-CoV-2, mucosal vaccines, adenoviruses

## Abstract

The COVID-19 pandemic has emphasized the importance and need for accessible safe, effective, and versatile vaccine platforms. While approved SARS-CoV-2 vaccines have been instrumental in saving lives and reducing healthcare and economic burdens, the induction of mucosal immunity remains an unmet need. Here, we engineered and evaluated a non-replicating adenovirus 5 (rAd5)-based vaccine expressing the SARS-CoV-2 S1 subunit (rAd5-SARS2-S1). We assessed the immunogenicity, durability, and protective efficacy of intramuscular (IM) and intranasal (IN) administration of rAd5-SARS2-S1 in mice and Syrian hamsters. Two IM or IN doses of rAd5-SARS2-S1 elicited robust and sustained Th1-skewed S1-specific serum IgG, neutralizing antibodies (nAbs) against several SARS-CoV-2 variants and systemic antigen-specific memory T cell responses in mice. Additionally, IN vaccination induced potent and long-lasting mucosal S1-specific IgG, IgA, and nAbs and pulmonary memory T cells. Importantly, while IM vaccine significantly ameliorated disease severity in hamsters by reducing viral burden, lung pathology, and, to some extent, weight loss, IN immunization significantly reduced viral replication and provided superior protection against disease and weight loss. Together, our study demonstrates that the rAd5-SARS2-S1 vaccine is immunogenic in both mice and hamsters when administered intramuscularly or intranasally, with IN administration providing better protection. These findings suggest that IN delivery of rAd5-SARS2-S1 could be a promising approach for inducing mucosal and systemic immunity, offering enhanced protection against SARS-CoV-2 and emerging variants.

**IMPORTANCE:**

This publication presents an assessment of the immune response and effectiveness of a vaccine containing genetically modified non-replicating recombinant that expresses the S1 subunit protein of SARS-CoV-2. We conducted a comparative analysis of the immune response potency, durability, and protective effectiveness of this vaccine using intramuscular (IM) and intranasal (IN) inoculation in mice and Syrian hamsters. Our findings indicate that both vaccinations were effective in stimulating strong and long-lasting immune responses, both locally and across the body, when administered through either IM or IN methods. Crucially, our study demonstrated that the IN vaccination outperformed the IM vaccine by effectively and significantly suppressing the multiplication of the virus in the lungs and nasal turbinates. Additionally, the IN vaccine provided protection against disease-related weight loss and lung damage in the animals. This work showcases the potential of intranasal administration as a viable method to stimulate both mucosal and systemic immunity. This technique provides improved defense against SARS-CoV-2 and maybe additional variations.

## INTRODUCTION

Unprecedented time, effort, and resources have been put toward vaccine development against the severe acute respiratory syndrome coronavirus 2 (SARS-CoV-2). It is estimated that there are around 400 vaccine candidates in clinical or preclinical development worldwide, with at least 50 pre-qualified vaccines globally by the World Health organization (WHO) and/or approved by local health authorities in several countries (1). These vaccines utilize various approaches and platforms including attenuated viruses, inactivated viruses, viral vectored vaccines, recombinant protein-based vaccines, nucleic acid-based vaccines (mRNA or DNA), and virus-like particle (VLP) vaccines (1–5).

Licensed vaccines have been instrumental in the global fight against SARS-CoV-2 by limiting transmission and reducing disease severity and hospitalizations, preventing millions of deaths (6–8). However, some vaccines have shown limitations, particularly in inducing strong mucosal immunity (9–16). There is a need for vaccines that are both safe and capable of providing potent long-lasting immunity at mucosal surfaces where initial viral entry occurs.

While most COVID-19 vaccines have been approved for intramuscular (IM) administration, several intranasal (IN) adenovirus-based vaccines were developed and/or entered clinical trials (17–25). Despite the disappointing or unsatisfactory results of some of these candidates (20–22), at least two vaccines, namely, AD5-nCOV (CanSino) and BBV154 (Bharat), were licensed as IN vaccines in China and India, respectively (5, 26, 27). IN vaccines can induce local immunity at the initial sites of infection, potentially preventing infections and transmission more effectively than IM vaccines. Additionally, IN vaccines can be used as booster doses for IM vaccines in heterologous regimens to further enhance both systemic and local immune responses (17, 20, 28–31). Therefore, IN vaccines could be a valuable addition to the current vaccine arsenal to combat future viral infections with pandemic potential.

Most IM and IN SARS-CoV-2 vaccines especially those based on viral vectors such as adenovirus target the full spike (S) glycoprotein or the receptor-binding domain (RBD) within the S1 subunit to prevent the attachment of the viral RBD to the host cell receptor angiotensin-converting enzyme 2 (ACE2) via the induction of neutralizing antibodies (nAbs) (32). Previous studies on SARS-CoV-1 and MERS-CoV also demonstrated the potential immunogenicity and protective efficacy of these target proteins (2, 33). However, the use of full-length S protein induces non-nAbs which have been linked to risks of antibody-dependent enhancement (ADE) in MERS-CoV and SARS-CoV-1 (34–36). Notably, while ADE has not been observed clinically with the use of SARS-CoV-2 vaccines, poor or suboptimal nAbs response has been linked to increased disease severity COVID-19 patients (37). Therefore, we chose to use the S1 subunit of the protein not only to focus the immune response on the neutralizing epitope-rich regions such as the RBD and the N-terminal domain (NTD) to elicit protective immunity but also to help minimize the potential risks of ADE due to non-nAbs.

In this study, we genetically engineered recombinant adenovirus type 5 (rAd5) to express secreted SARS-CoV-2 S1 subunit protein (rAd5-SARS2-S1). We compared IM and IN administration of this vaccine to a vector control expressing green fluorescent protein (rAd5-GFP) in mice and hamsters. We assessed humoral and cellular immunity in mice and evaluated protective efficacy in Syrian hamsters. Our results showed that both IM and IN immunization with rAd5-SARS2-S1 induced strong immunity in mice and provided better protective efficacy when administered intranasally in Syrian hamsters. These findings suggest that this vaccine platform is a promising candidate for further development.

## MATERIALS AND METHODS

### Immunogen *in silico* design and generation of the rAd5-based vaccines

The SARS-CoV-2 S1 gene was designed to express a secreted S1 protein (amino acids 1–681) from the ancestral Wuhan strain (GenBank accession number: MN908947). The gene was codon optimized for mammalian expression using the implemented application in Geneious Prime software version 2020.1.2 (https://www.geneious.com). The signal peptide from SARS-CoV-2 S protein was maintained at the N-terminus to facilitate protein secretion from rAd5-infected cells. This synthetic gene was commercially synthesized (Genscript, Piscataway, NJ, USA) and used to generate the recombinant Ad5 construct (rAd5-SARS2-S1) under the regulation of the Cytomegalovirus (CMV) promoter. An empty control vector expressing GFP (rAd5-GFP) was also used (26). AdEasy Adenoviral Vector System (Agilent, Santa Clara, CA, USA) was used to generate recombinant Ads according to the manufacturer’s instructions in human embryonic kidney (HEK-293A) cells using Lipofectamine 2000 transfection reagent (Invitrogen, Carlsbad, CA, USA). Recombinant adenoviruses were amplified in HEK-293A cells, purified using a sucrose cushion, and titrated using Adeno-X rapid titer kit (Takara Bio, San Jose CA, USA).

### Cell lines and viruses

Human embryonic kidney HEK293A, Baby Hamster kidney BHK-21/WI-2 (Kerafast, EH1011) cells, and African Green monkey kidney-derived Vero E6 (ATCC, 1586) cells were cultured in Dulbecco’s modified Eagle medium (DMEM) supplemented with 25 mM HEPES, 1× non-essential amino acid, 20 U/mL Penicillin, 0.02 mg/mL Streptomycin, 1 mM sodium pyruvate, and 10% heat-inactivated fetal bovine serum (FBS). SARS-CoV-2 isolate Canada/ON/VIDO-01/2020 was propagated in Vero E6 cells, titrated on Vero E6 cells, and sequenced to confirm the genetic sequence. Passage three virus stocks were used in all subsequent experiments that required live virus.

### Plasmids

Plasmids expressing codon-optimized truncated S protein (lacking the last 21 residues at the C-terminal) from the ancestral Wuhan SARS-CoV-2 strain (GenBank accession number: MN908947) were previously generated (36, 38). Variants including 614G, Alpha (B.1.1.7), Beta (B.1.351), Gamma (P.1), and Delta (B.1.617) were generated from the plasmid expressing ancestral Wuhan SARS-CoV-2 strain as a template for site-directed mutagenesis using In-Fusion HD Cloning plus (Takara Bio, San Jose CA, USA) to introduce the needed mutations as previously described (39). All constructs were confirmed by sequencing.

### Animal study

The vaccine candidate was tested in several animal experiments using 8- to 10-week-old female Balb/c mice and Syrian hamsters. Balb/c mice were obtained from King Fahd Medical Research Center (KFMRC), King Abdulaziz University, while the Syrian hamsters were purchased from Charles River Laboratories (Saint-Constant, Canada). All animal work was conducted following the guidelines of the Animal Care and Use Committee (ACUC) at KFMRC. All animals were randomly allocated into different groups in each experiment and received two doses of the vaccine or control (50 µL per dose) on days 0 (prime) and 21 (boost) via IM or IN route. The first experiment was a dose escalation study (*n* = 7 mice per group) to determine the optimal vaccine dose by testing three different doses (1 × 10^7^, 1 × 10^8^, and 1 × 10^9^ PFU) administered intramuscularly. Blood was collected on days 0, 21, 42, and 90 via retro-orbital bleeding to evaluate the humoral response. The second experiment was to study the immunogenicity of IM immunization of mice with 1 × 10^9^ PFU dose of the vaccine (*n* = 5 mice per group). Blood was collected on days 0, 21, 42, and 90 to evaluate the humoral response. The third experiment was to study the immunogenicity of IN immunization of mice with 1 × 10^9^ PFU dose of the vaccine (*n* = 10 mice per group). Blood and bronchoalveolar lavage fluid (BALF) were collected on days 42 and 90 to evaluate the humoral response in each group, in which 5 mice were euthanized on day 42 and day 90 to collect BALF. The fourth experiment was done in Syrian hamsters to evaluate the protective efficacy of the vaccine. Hamsters were immunized as indicated above, and blood samples were collected on days 14 and 35. On day 49 post-vaccination, the hamsters were intranasally challenged with 1.0 × 10^5^ PFU of SARS-CoV-2 (Canada/ON/VIDO-01/2020). Hamsters were observed daily for signs of morbidity or mortality. No deaths were observed post-inoculation. Furthermore, every possible measure was implemented to prevent any form of animal distress at every phase of this experiment. Animals were euthanized by CO_2_ either 3- or 6-days post-infection (dpi) and nasal turbinate and lung tissues were collected for downstream experiments. The study was approved by CEGMR bioethics committee at King Abdulaziz University (approval number 04-CEGMR-Bioeth-2020), and the animal study protocol was approved by King Abdulaziz University Animal Care and Use Committees (approval number ACUC-20-03-9), with all infectious materials handled at BSL-3 facilities.

### Enzyme-linked immunosorbent assay

Detection of total binding IgG, its isotypes (IgG1, IgG2a, and IgG2b), and IgA in sera after IM and IN immunization, as well as IgA in sera and BALF after IN vaccination, against ancestral SARS-CoV-2 S1 subunit (amino acids 1–685) or receptor-binding domain (RBD) (amino acids 319–541) was performed by indirect enzyme-linked immunosorbent assay (ELISA) as previously described (40, 41). Briefly, recombinant S1 and RBD proteins (Sino Biological, China) were used to coat 96-well high-binding ELISA plates (Greiner Bio One, Monroe, NC, USA) at 1 µg/mL in 1× PBS buffer (pH 7.4) with 50 µL per well for overnight incubation at 4°C. Plates were washed three times with 1× PBS containing 0.05% Tween 20 (PBS-T) and blocked with 5% skim milk in PBS-T buffer (blocking buffer) at room temperature for 2 h. After washing, twofold serially diluted sera (100 µL/well) starting from a 1:100 dilution in blocking buffer were added and incubated for 1 h at 37°C. Some samples were only tested at a 1:100 dilution. Then, HRP-conjugated goat anti-mouse IgG, IgG1, IgG2a, or IgG2b antibodies, anti-Syrian Hamster IgG (Jackson ImmunoResearch, Cambridge, UK), or goat anti-mouse IgA (Southern Biotech, Birmingham, AL, USA) were added as secondary antibodies at manufacturer-recommended dilutions in blocking buffer and incubated for 1 h at 37°C. Plates were washed three times, incubated with 3,3′,5,5′-tetramethylbenzidine (TMB) substrate (KPL, Gaithersburg, MD, USA) at room temperature for 15 min in the dark, and the reaction was stopped by 0.16 M sulfuric acid. Optical density (OD) was spectrophotometrically measured at 450 nm using BioTek Synergy 2 microplate reader (BioTek, Winooski, VT, USA). Endpoint titers were calculated and expressed as the reciprocals of the highest dilutions with OD readings at 450 nm that are greater than the cut-off value, defined as the mean OD value of the serum from control animals + 3 times the standard deviation (SD).

### Pseudovirus-based microneutralization assay

The SARS-CoV-2-pseudovirus neutralization assay was conducted using recombinant vesicular stomatitis virus (rVSV)-based pseudovirus as previously described (38, 42). Briefly, rVSV pseudoviruses expressing SARS-CoV-2 S protein from the ancestral Wuhan strain or 614G, alpha, beta, gamma, or delta variants were generated in BHK21/WI-2 cells transfected with plasmids expressing corresponding proteins using lipofectamine 2000 (Invitrogen, Carlsbad, CA, USA). Transfected cells were then infected with rVSV-G/G*-luciferase 24 h after transfection. One hour after infection and incubation at 37°C, the virus inoculum was removed, and cells were washed and incubated in complete 5% DMEM containing rabbit polyclonal anti-VSV-G antibody at 1:1,000 for 24 h at 37°C and 5% CO_2_. Supernatants containing the rVSV pseudoviruses were collected 24 h after infection, titrated by measuring luciferase activity in infected Vero E6 cells, and expressed as relative luciferase units (RLU). The neutralization assay was performed by incubating equal volumes of twofold serially diluted BALF or heat-inactivated sera, prepared in DMEM with 5% FBS, starting from 1:20 dilution with DMEM containing 5 × 10^4^ RLU of each pseudovirus for 1 h at 37°C in a 5% CO_2_ incubator. Subsequently, 100 µL of the mixture was transferred to white 96-well plates containing confluent Vero E6 cells and incubated for 24 h at 37°C in 5% CO_2_. Each dilution was tested in duplicate. After 24 h incubation, media were removed, and cells were washed with PBS and lysed by 1× cell culture lysis buffer. Luciferase activity was measured as RLU using BioTek Synergy 2 microplate reader (BioTek, Winooski, VT, USA) as previously described (39). Each assay run included a cell-only control (CC) and a virus control (VC). The inhibition of luciferase activity by each dilution was calculated according to the following equation: 100 – [(average RLU from each dilution – average RLU from CC)/(average RLU from VC – average RLU from CC) × 100]. Then, neutralization titers were computed as half maximal inhibitory concentration (IC_50_) using four-parameter logistic (4PL) curve in GraphPad Prism V9 software (GraphPad Co., San Diego, CA, USA).

### T cell intracellular cytokine staining

Spleens from mice immunized intramuscularly, and spleens and lungs from mice immunized intranasally (*n* = 5) with two doses of 1 × 10^9^ PFU of rAd5-SARS2-S1or rAd5-GFP were harvested on day 90. Spleens were processed as described previously (43, 44). Lungs were cut into small pieces and incubated at 37°C with an extraction buffer (5 mL RPMI with 2 mg/mL collagenase D) for 1 h with agitation. Then, digested lung tissues were mashed, passed through 70 µm cell strainer, washed with PBS, and spun down at 500 × *g* for 5 min. The pellet was carefully overlayed with 67.5% and 45% percoll and centrifuged at 800 × *g* for 15 min at room temperature. Lymphocytes were carefully collected from the band formed at 45% and 67.5% interface, transferred to a sterile tube, washed twice with PBS, and centrifuged at 800 × *g* for 5 min. Red blood cells (RBCs) were lysed with 1× ammonium-chloride-potassium (ACK) lysis buffer (1.5M NH4Cl, 100 mM NaHCO3, and 10 mM Na-EDTA, pH adjusted to 7.4 with 6N HCl) incubated for 3 min at room temperature. Then, cells were centrifuged at 800 × *g* for 5 min and washed with PBS. Single-cell suspensions of splenocytes and lung lymphocytes were prepared from each individual animal at a concentration of 1 × 10^7^ cells/mL in complete RPMI-1640 media supplemented with 10% FBS. Around 1 × 10^6^ cells from single-cell suspensions of splenocytes or lung lymphocytes were transferred to 96-well round plate and centrifuged at 400 × *g* for 5 min at room temperature. Supernatants were discarded, and cell pellets were *ex vivo* re-stimulated with pool of 15-mer peptides overlapping by 11 residues (5 ug/mL) covering SARS-CoV-2 S1 subunit protein (Genscript USA Inc., Piscataway, NJ) in complete RPMI-1640 media supplemented with 10% FBS for 6 h at 37°C. Negative control cells were stimulated with an equal volume of 1% dimethylsulfoxide (DMSO) in RPMI 1640 medium. Positive control cells were stimulated with phorbol 12-myristate 13-acetate (PMA, 0·5 mg/mL) and ionomycin (1 mg/mL). After 1 h of re-stimulation, Protein Transport Inhibitor Cocktail-brefeldin A (BD Biosciences, San Jose, CA, USA) was added at 1:1,000 to each sample. After 6 h, cells were washed with PBS and stained with a LIVE/DEAD Fixable Near-IR Dead Cell Stain Kit (Invitrogen, Carlsbad, CA, USA) for 30 min at room temperature in the dark. After washing with fluorescence-activated cell sorting (FACS) buffer (PBS with 2% heat inactivated FBS), cells were stained with the following surface staining anti-mouse antibodies cocktail diluted in FACS buffer: CD8-pacific Blue (clone 53-5.8), CD4-PE/Dazzle594 (clone RM4-5), CD44-APC (clone IM7), and CD62L-PE-Cy7 (clone MEL-14). Cells were stained for 20 min at 4°C in the dark. Then, the cells were washed with FACS buffer and centrifuged at 400 × *g* for 5 min at room temperature, fixed and permeabilized using Cytofix/Cytoperm Solution (BD Biosciences, San Jose, CA, USA) according to the manufacturer’s protocol. For intracellular staining, cells were stained with the following anti-mouse antibodies cocktail diluted in perm wash buffer for 30 min at 4°C in the dark: IFNγ-FITC (clone XMG1.2), TNFα-PE (clone MP6-XT22), and IL2-Per-Cy5.5 (clone JES6-5H4). Cells were then washed twice with permeabilization buffer and once with FACS buffer, and supernatants were discarded, and washed cell pellets were resuspended in 200 µL/well of FACS buffer and then transferred to FACS tubes for analysis. All data were acquired using BD FACSAriaTM III flow cytometer (BD Biosciences, San Jose, CA, USA) and analyzed using FlowJo v10 software (Tree Star Inc., Ashland, OR, USA). All flowcytometry antibodies were purchased from BioLegend, San Diego, CA, USA.

### Lung viral titer

Plaque assays were performed as previously described (45). Briefly, lung and nasal turbinate tissues were mechanically homogenized in PBS. A 1:10 serial dilution of clarified supernatant was prepared in infection media (DMEM supplemented with 1× non-essential amino acid, 20 U/mL penicillin, 0.02 mg/mL streptomycin, 1 mM sodium pyruvate, and 0.1% bovine serum albumin). Virus was adsorbed on Vero cells at 37°C and 5% CO_2_ for 1 h replacing the inoculum with overlay media (1× infection media with 0.6% ultrapure and low-melting point agarose). Cells were incubated at 37°C and 5% CO_2_ for 72 h, fixed with 10% formaldehyde, and then stained with crystal violet. Plaques were enumerated and PFU was determined per gram of tissue.

### Quantitative real-time-PCR assay

SARS-CoV-2 E subgenomic mRNA (sgmRNA) levels in lungs and nasal turbinates were assessed by real-time-PCR (RT-qPCR) as previously described (45). Briefly, lung and nasal turbinate tissues were placed into RNA shield buffer (Zymo Research, Irvine, CA) and incubated overnight at 4°C before freezing at −80°C. Viral RNA was extracted in BSL-3 using mechanical homogenization and Quick-RNA Viral Kit (Zymo Research, Irvine, CA). Viral RNA expression was quantified using a one-step Fast Virus master mix (ThermoFisher, Ottawa, ON) according to the manufacturer’s protocol and E sgmRNA-specific primer/probe set (Table 1). Standard curves of *in vitro* transcribed SARS-CoV-2 E sgmRNA from a pcDNA3.1 plasmid were used as a standard to calculate sgmRNA copy numbers normalized by tissue weight. RT-qPCR reactions were conducted using an Applied Biosystems7500 Fast Real-time PCR instrument.

**TABLE 1:** E sgmRNA primers and probe

Name	Sequence
Forward primer	5′- CGATCTCTTGTAGATCTGTTCTC- 3′
Reverse primer	5′- ACACTAGCCATCCTTACTGCGCTTCG- 3′
Probe	5′- FAM-ATATTGCAGCAGTACGCACACA-MGB- 3′

### Histopathology

Histopathology analysis was conducted as described previously (45). Briefly, right lung lobes were fixed for 72 h in 10% neutral buffered formalin and embedded using paraffin. Then, 4 µm thick sections were prepared, stained with hematoxylin-eosin (HE), and examined under microscopy. The severity and extent of pneumonia were scored blinded based on previously established criteria (45, 46) as shown in Table 2.

**TABLE 2:** Pathology score criteria

Score	Histological changes
0	No significant finding
1	Minor peribronchial/bronchiolar and perivascular inflammation with slight thickening of alveolar septa with small numbers of mononuclear cell infiltration
2	Apparent inflammation and alveolus septa thickening with more interstitial mononuclear inflammatory infiltration; focal areas of consolidation
3	Multiple focal consolidation with alveolar septa thickening and increased infiltration of inflammatory cells
4	Area of consolidation with extensive alveolar septa thickening, collapse of alveoli, restricted fusion of the thick septa, and more cell infiltration in alveolar space and the areas surrounding airways and blood vessels
5	As 4, but the lung is almost completely consolidation

### Statistical analysis

Statistical parameters including standard deviations, means, *P*-values, and types of statistical tests are reported in the graphs and corresponding legends. GraphPad Prism software version 9.0.0 (GraphPad Co., San Diego, CA, USA) was used for data analysis and graphical presentations. Mann–Whitney test, one-way analysis of variance with Tukey’s test, or two-way analysis of variance was used for comparison between groups. All values are expressed as mean ± SD, and statistical significance is reported as *, *P* ≤ 0.05, **, *P* ≤ 0.01, ***, *P* ≤ 0.001, and ****, or *P* ≤ 0.0001.

## RESULTS

### SARS-CoV-2 S1 vaccine elicits strong and long-lasting systemic humoral immune response in mice upon intramuscular immunization

**Fig 1:**
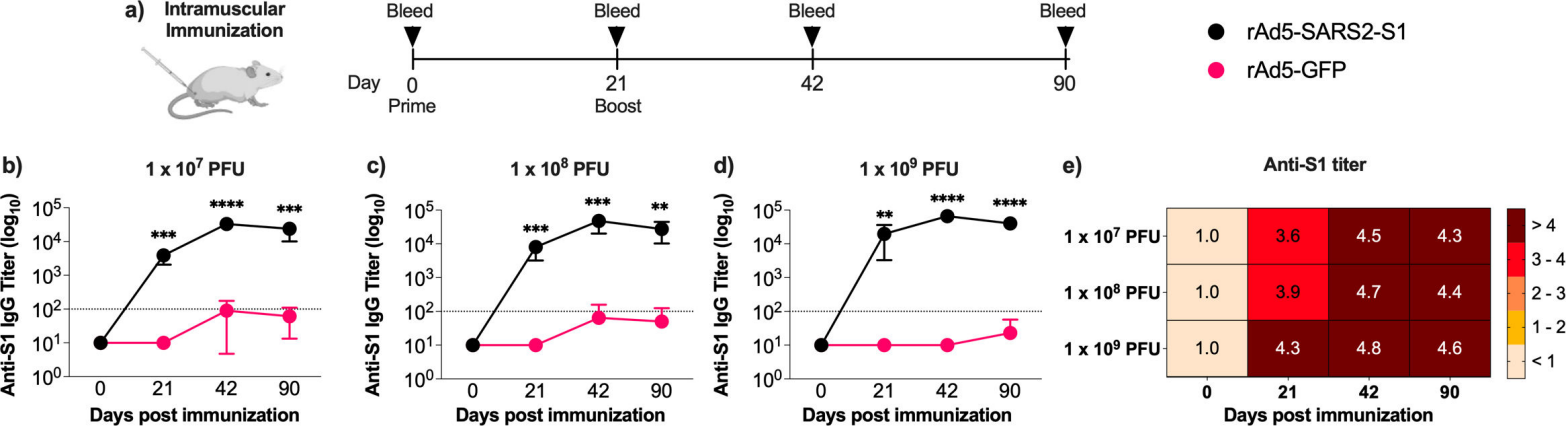
Vaccine dose escalation study. (**a**) Female Balb/c mice were randomly divided into three experimental groups (*n* = 7) and immunized intramuscularly on days 0 and 21 with one of three different doses (1 × 10^9^, 1 × 10^8^, and 1 × 10^7^ PFU) of rAd5-SARS2-S1 or rAd5-GFP. Blood samples were collected on days 0, 21, 42, and 90. Endpoint titers of S1-specific total binding IgG were determined by ELISA from immunized mice on days 0, 21, 42, and 90. Comparison of mean end-point titers of S1-specific total binding IgG elicited by rAd5-SARS2-S1 or rAd5-GFP using (**b**) 1 × 10^7^ PFU, (**c**) 1 × 10^8^ PFU, and (**d**) 1 × 10^9^ PFU doses. (**e**) Heatmap showing the S1-specific binding IgG endpoint titer elicited by each dose of rAd5-SARS2-S1 over time. Data are presented as mean ± SD. Statistical significance was determined by Mann–Whitney test in panels (**a–c**) and reported as **, *P* < 0.01; ***, *P* < 0.001; ****, *P* < 0.0001. Dotted lines represent cutoffs of the assays.

To determine the optimal dosage of the generated rAd5-based SARS-CoV-2 vaccine, 6–8 week-old female Balb/c mice were immunized intramuscularly with two doses of rAd5-SARS2-S1 or rAd5-GFP on days 0 and day 21 using one of three different doses 1 × 10^9^, 1 × 10^8_,_^ or 1 × 10^7^ PFU. Sera were collected on days 0, 21, 42, and 90 and tested for S1-specific total binding IgG (Fig. 1a). The rAd5-SARS2-S1 vaccine elicited strong and significant total IgG titer at all doses after the first dose compared to the rAd5-GFP control. The second dose further increased IgG levels as shown on days 42 and 90 (Fig. 1b through d) with the highest levels of S1-specific total IgG observed at the highest dose of the vaccine (1 × 10^9^PFU) which maintained durable antibody responses that lasted for up to 90 days (Fig. 1e). Thus, two doses of 1 × 10^9^PFU were selected as the immunization regimen in subsequent studies.

**Fig 2:**
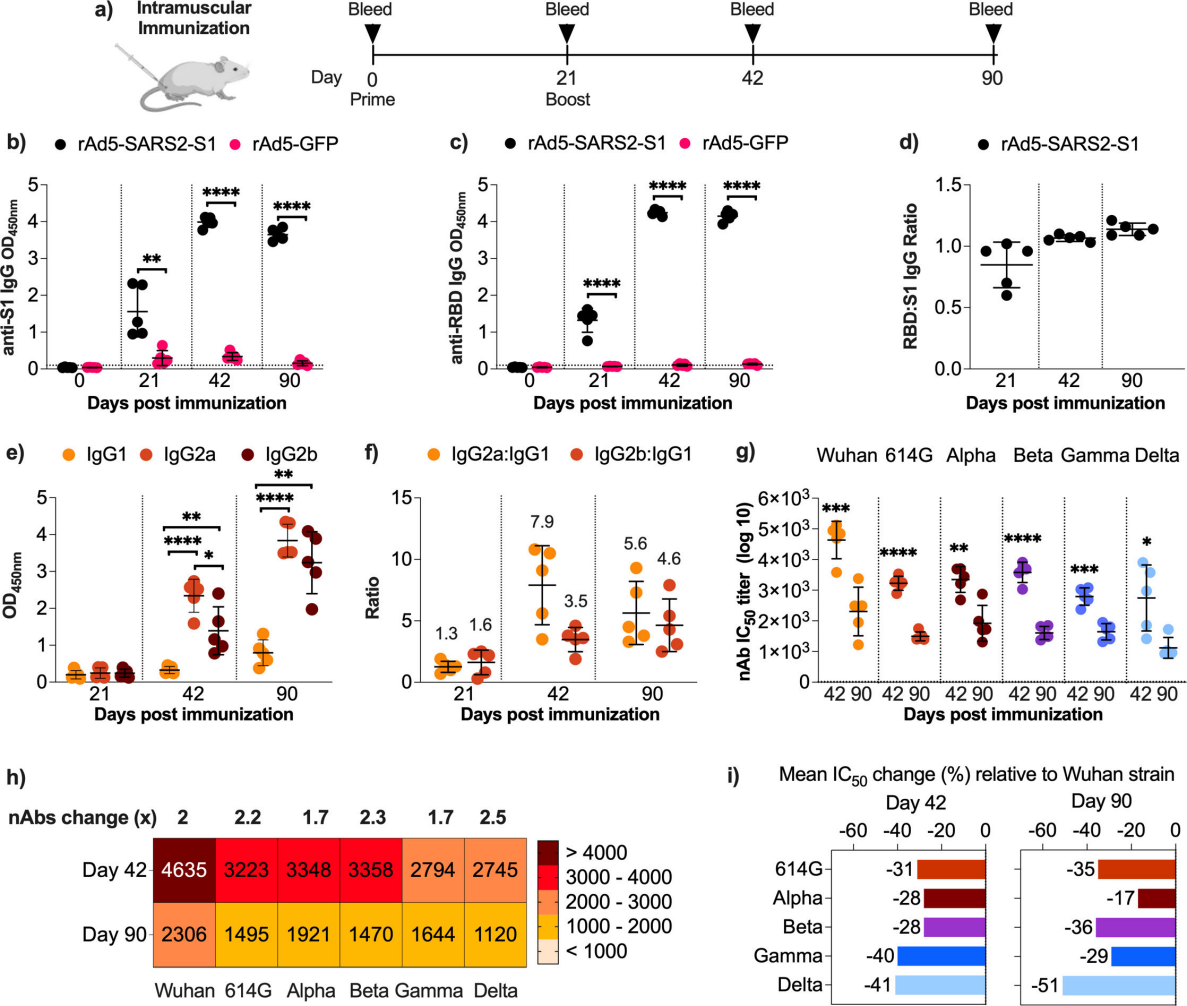
Humoral immune response induced by intramuscular immunization. (**a**) Female Balb/c mice were randomly divided into two experimental groups (*n* = 5) and immunized intramuscularly on days 0 and 21 with 1 × 10^9^ PFU of rAd5-SARS2-S1 or rAd5-GFP. Blood samples were collected on days 0, 21, 42, and 90. Mean optical density (OD) values of (**b**) S1- and (**c**) RBD-specific total binding IgG at 1:100 dilution from immunized mice were determined by ELISA on days 0, 21, 42, and 90. (**d**) Ratio (OD values) of anti-RBD:anti-S1 total binding IgG from mice immunized with rAd5-SARS2-S1 on days 21, 42, and 90. Mean OD values of S1-specific IgG1, IgG2a, and IgG2b at 1:100 dilution from sera obtained from mice immunized with rAd5-SARS2-S1 (**e**) and ratio (OD values) of IgG2a:IgG1 and IgG2b:IgG1 (**f**) as determined by ELISA on days 21, 42, and 90. (**g**) The IC_50_ of nAbs in sera obtained on days 42 and 90 from mice immunized with two doses of rAd5-SARS2-S1 vaccine as determined against pseudoviruses expressing spike protein from ancestral Wuhan strain and D614G, Alpha, Beta, Gamma, and Delta variants. (**h**) Heatmap showing the nAbs IC_50_ values of sera against the different SARS-CoV-2 pseudoviruses on days 42 and 90 in sera. Numbers above the heatmap show the fold reduction in mean IC_50_ from day 42 to day 90 in sera. (**i**) Change (%) in mean IC_50_ from sera obtained on days 42 and 90 from mice immunized with rAd5-SARS2-S1 against each SARS-CoV-2 variant relative to the ancestral Wuhan SARS-CoV-2 strain. Data are shown as the mean ± SD. Statistical significance was determined by Mann–Whitney test in panels (**b, c, and g**), and one-way ANOVA analysis of variance with Tukey’s *post hoc* test in panel (**e**). Significance was reported as *, *P* < 0.05; **, *P* < 0.01; ***, *P* < 0.001; ****, and *P* < 0.0001. Dotted lines represent cutoffs of the assays.

Next, we further evaluated the immunogenicity of rAd5-SARS2-S1 vaccine. Female Balb/c mice were immunized intramuscularly with two doses of 1 × 10^9^ PFU of the vaccine (Fig. 2a), and sera were collected and tested for S1- (Fig. 2b) and RBD- (Fig. 2c) specific total binding serum IgG on days 0, 21, 42, and 90 using an indirect ELISA. The rAd5-SARS2-S1 vaccine induced durable and significant systemic levels of both S1- and RBD-specific IgG that lasted up to 90 days compared to rAd5-GFP (Fig. 2b and c). Following the boost vaccination S1- and RBD-specific IgG levels at day 42s and 90 greatly increased relative to levels on day 21 (Fig. 2b and c). Comparing RBD:S1 IgG levels showed that both antibody types induced by the vaccine were similar (ratio of ~1) at all time points (Fig. 2d), indicating that most of the antibodies induced by rAd5-SARS2-S1 recognized the RBD in the S1 subunit. As expected, the control vector (rAd5-GFP) failed to elicit any S1- or RBD-specific antibody response. In addition, collected sera were tested for S1-specific IgG subclasses (IgG1, IgG2a and IgG2b) as a surrogate marker for the type of induced immune response (Th1 and Th2) on days 21, 42, and 90 (Fig. 2e and f). The vaccine elicited significantly higher levels of IgG2a and IgG2b compared to IgG1 after boosting (Fig. 2e) as reflected in the >1 IgG2a:IgG1 and IgG2b:IgG1 ratios (Fig. 2f), suggesting a Th1-skewed humoral immune response. The booster dose of the vaccine significantly elevated the levels of IgG2a and IgG2b antibodies with some increase in IgG1 by day 90 (Fig. 2e). Together, these data indicate that S1-based rAd5 vaccine resulted in RBD-focused and Th1-skewed immune responses.

**Fig 3:**
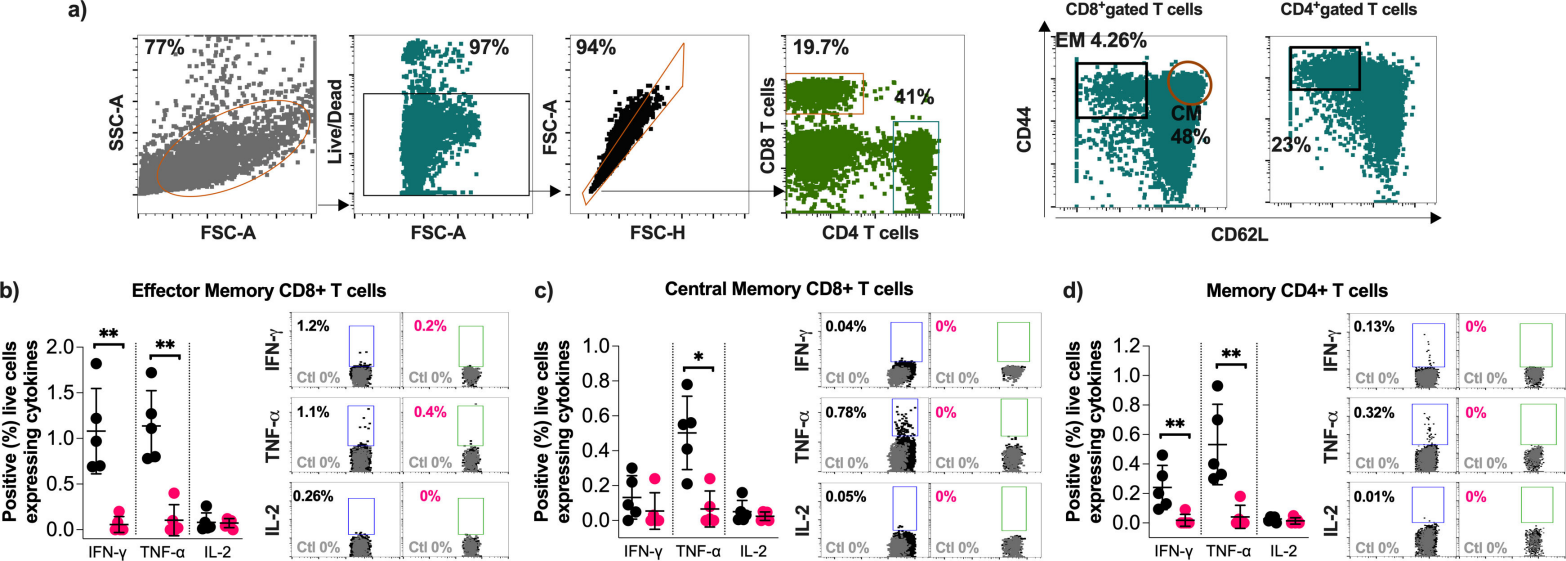
Memory T cells immune response induced by intramuscular immunization. Female Balb/c mice were randomly divided into four experimental groups (*n* = 5) and immunized intramuscularly on days 0 and 21 with 1 × 10^9^ PFU of rAd5-SARS2-S1 or rAd5-GFP. Animals were euthanized on day 90. Splenocytes were isolated and *ex vivo* re-stimulated with a synthetic peptide pool covering the ancestral Wuhan SARS-CoV-2 strain S1 protein for 6 h. (**a**) Representative FACS plots of the gating strategies on live effector CD8^+^CD44^hi^CD62L^−^ memory T cells, central CD8^+^CD44^hi^CD62L^+^ memory T cells, and memory CD4^+^CD44^hi^CD62L^−^ T cells. Histograms and representative FACS plots displaying the percentages of IFN-γ^+^, TNF-α^+^, and IL-2^+^ (**b**) effector memory CD8^+^ T cells, (**c**) central memory CD8^+^ T cells, and (**d**) memory CD4^+^ T cells. All the represented data were calculated by subtracting the values of the expressed levels by non-stimulated cells (ctl) from the re-stimulated cells. Results are represented as mean ± SD. Statistical significance was determined by Mann–Whitney test and reported as *, *P* < 0.05; **, *P* < 0.01.

To further extend our analysis, nAbs levels in the sera were evaluated against ancestral Wuhan strain and D614G, Alpha, Beta, Gamma, and Delta variant pseudoviruses on days 21, 42, and 90 (Fig. 2g through i). While a single dose of rAd5-SARS2-S1 (i.e., day 21) elicited higher nAbs against the ancestral Wuhan strain than the rAd5-GFP control, the levels were low (data not shown). However, more robust nAb responses were induced and maintained following the boost vaccination (Fig. 2g), indicating that at least two doses of rAd5-SARS2-S1 are required to induce high nAb titers. Moreover, the vaccine exhibited strong neutralizing responses against all tested variants after two doses (Fig. 2g), with nAb levels being maintained until day 90 despite significant waning ranging from 1.7- to 2.5-fold reduction (43%–59% reduction) (Fig. 2h). We further compared the % change (loss or gain) in mean IC_50_ values against SARS-CoV-2 variants relative to the ancestral Wuhan strain (Fig. 3i). Unsurprisingly, nAbs titers against all variants declined in rAd5-SARS2-S1 vaccinated group. This loss of neutralizing activity ranged between 28%–41% and 17%–51% on days 42 and 90, respectively (Fig. 2i). These findings clearly confirm that this ancestral Wuhan strain-based rAd5-SARS2-S1 vaccine is immunogenic when given intramuscularly and able to elicit nAbs against several variants.

### Intramuscular immunization with rAd5-SARS2-S1 elicits long-lasting memory T cell immune responses in mice

The observed Th1-biased humoral response in immunized mice prompted us to evaluate systemic memory T cell immune responses in female Balb/c mice immunized intramuscularly with two doses (1 × 10^9^ PFU) of rAd5-SARS2-S1 and rAd5-GFP. Splenocytes isolated from immunized mice on day 90 were re-stimulated *ex vivo* with a synthetic peptide pool covering the entire ancestral Wuhan SARS-CoV-2 strain S1 subunit protein. After re-stimulation, S1-specific live IFN-γ, TNF-α, and IL-2 cytokines secreting effector CD8^+^CD44^hi^CD62L^−^ memory T cells (CD8^+^ TEM), central CD8^+^CD44^hi^CD62L^+^ memory T cells (CD8^+^ TCM), and memory CD4^+^CD44^hi^CD62L^−^ T cells were evaluated (Fig. 3a). As shown in Fig. 3b, rAd5-SARS2-S1 immunized mice showed significant levels of IFN-γ^+^ and TNF-α^+^ but not IL-2^+^ CD8^+^ TEM cells compared to the control group. Also, significant levels of TNF-α^+^ but not IFN-γ^+^ nor IL-2^+^ CD8^+^ TCM cells were only found in rAd5-SARS2-S1 immunized mice (Fig. 3c). Examining memory CD4^+^ T cells showed that the rAd5-SARS2-S1 vaccinated group produced significantly higher numbers of IFN-γ^+^ and TNF-α^+^ but not IL-2^+^ memory CD4^+^ T cells relative to the rAd5-GFP group (Fig. 3d). These data showed that the rAd5-SARS2-S1 vaccine elicited type I cytokine-producing memory CD8^+^ and CD4^+^ T cells in mice.

### Intranasal rAd5-SARS2-S1 vaccine induces strong and durable systemic and local humoral immunity in mice

**Fig 4:**
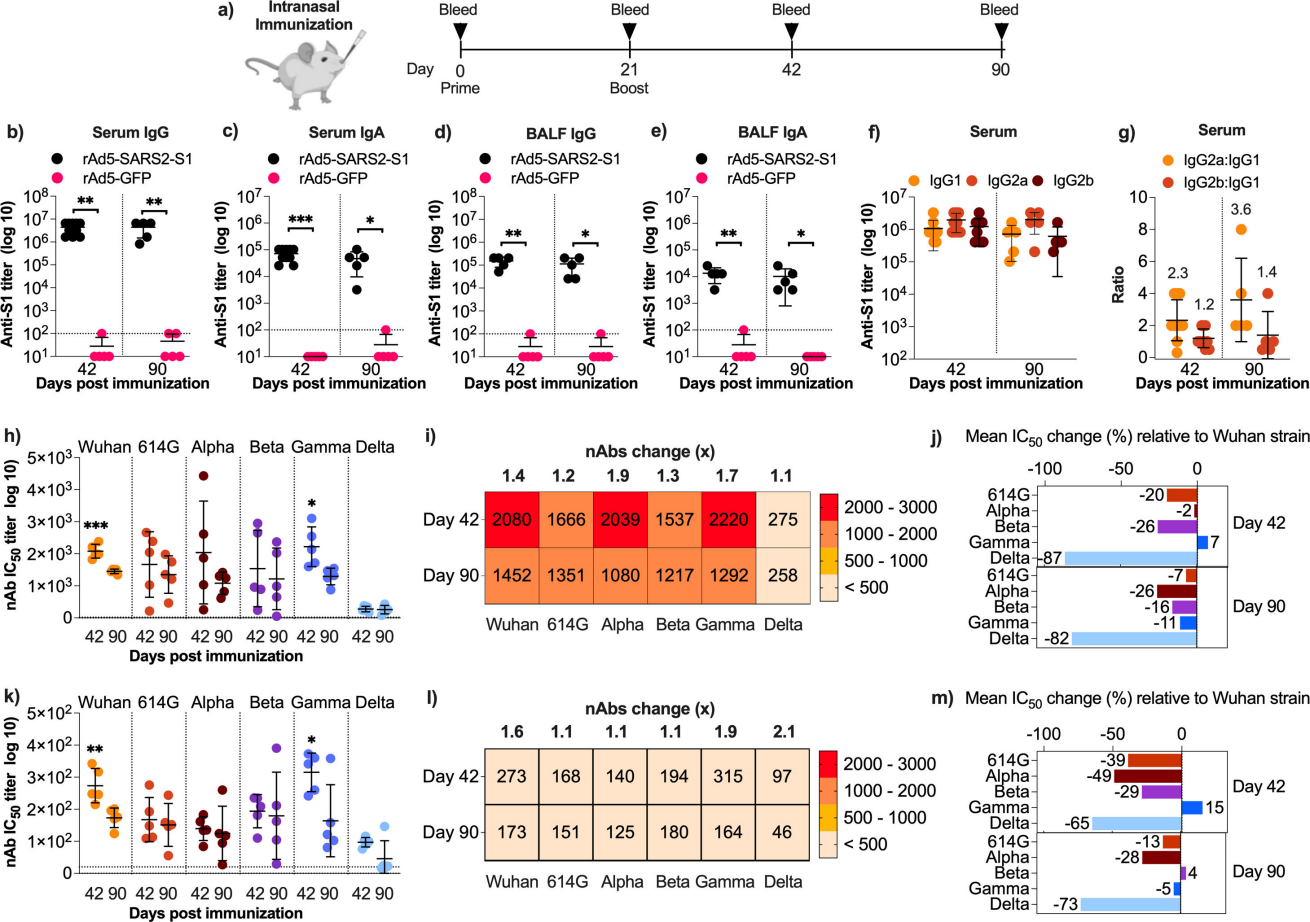
Humoral immune response induced by intranasal immunization. (**a**) Female Balb/c mice were randomly divided into two experimental groups (*n* = 10) and immunized intranasally on days 0 and 21 with 1 × 10^9^ PFU of rAd5-SARS2-S1 or rAd5-GFP. Blood and BALF samples were collected from mice (*n* = 5 per time point) euthanized on days 42 and 90. Endpoint titers of S1-specific binding serum (**b**) IgG and (**c**) IgA, and BALF (**d**) IgG and (**e**) IgA from mice immunized with rAd5-SARS2-S1 or rAd5-GFP as determined by ELISA on days 42 and 90. (**f**) Endpoint titers of S1-specific binding IgG1, IgG2a, and IgG2b from sera obtained from rAd5-SARS2-S1 immunized mice, and (**g**) ratio of IgG2a:IgG1 and IgG2b:IgG1 as determined by ELISA on days 42 and 90. The IC_50_ of nAbs in (**h**) sera and (**k**) BALF obtained on days 42 and 90 from mice immunized with two doses of rAd5-SARS2-S1 vaccine as determined against pseudoviruses expressing spike protein from ancestral Wuhan strain and D614G, Alpha, Beta, Gamma, and Delta variants. Heatmap showing the nAbs IC_50_ values of (**i**) sera and (**l**) BALF against the different SARS-CoV-2 pseudoviruses on days 42 and 90. Numbers above the heatmap show the fold reduction in mean IC_50_ from day 42 to day 90 (**i**) sera and (**l**) BALF. Change (%) in mean IC_50_ from (**j**) sera and (**m**) BALF obtained on days 42 and 90 from mice immunized with rAd5-SARS2-S1 against each SARS-CoV-2 variant relative to the ancestral Wuhan SARS-CoV-2 strain. Data are shown as the mean ± SD. Statistical significance was determined by Mann–Whitney test in panels (**b, c, d, e, h, and k**). Significance was reported as *, *P* < 0.05; **, *P* < 0.01; ***, *P* < 0.001; ****, and *P* < 0.0001. Dotted lines represent cutoffs of the assays.

To investigate the potential of our vaccine candidate to induce mucosal immunity, female Balb/c mice were vaccinated intranasally with two doses (1 × 10^9^ PFU) of rAd5-SARS2-S1 or rAd5-GFP on days 0 and 21 (Fig. 4a). Serum and BALF samples from the immunized mice were collected on days 42 and 90 (i.e., 3 and 10 weeks after boost) to evaluate S1-specific binding IgG and IgA levels as well as nAbs. The rAd5-SARS2-S1 vaccine induced a strong and durable systemic immune response with high levels of serum anti-S1 IgG and IgA that persisted for 10 weeks after boosting (Fig. 4b and c). Similar results were obtained in BALF antibody levels as immunized mice developed high levels of anti-S1 IgA and IgG (Fig. 3d and e). The vaccine elicited a systemic Th1-biased response based on the >1 IgG2a:IgG1 and IgG2b:IgG1 ratios on day 42 and day 90 (Fig. 4f and g). Notably, these ratios were lower than those observed with IM immunization. Higher levels of IgG1 were clearly induced following IN vaccination (titers ~ 10^6^) compared to IM administration (titers ~ 10^3^) (Fig. 2f).

Sera and BALF samples collected on days 42 and 90 were tested for their ability to neutralize pseudoviruses as described before. The titers of systemic nAbs elicited against SARS-CoV-2 and its variants by IN administration of rAd5-SARS2-S1 were as high as those induced following IM immunization except for the Delta variant (Fig. 4h and i). Specifically, while the vaccine induced very high levels of nAbs against all variants (>10^3^) on days 42 and 90, we observed ~1 log reduction (>80% loss of activity) in nAbs IC_50_ against the Delta variant when compared to neutralizing activity against the ancestral Wuhan strain (Fig. 4j). A similar trend in nAbs levels in the BALF was observed although titers were lower by ~1 log compared to levels seen in circulation (Fig. 4k through m). While serum and BALF nAbs titers declined over time, with 1.1–1.9- and 1.1–2.1-fold reductions respectively, this waning was mostly minimal except for few strains (Fig. 4h and i, k and l). Together, the IN administration of rAd5-SARS2-S1 demonstrated the ability to induce durable systemic and local IgG, IgA, and nAbs in mice.

### Intranasal immunization with rAd5-SARS2-S1 elicits potent and long-lasting systemic and local T cell immune response in mice

**Fig 5:**
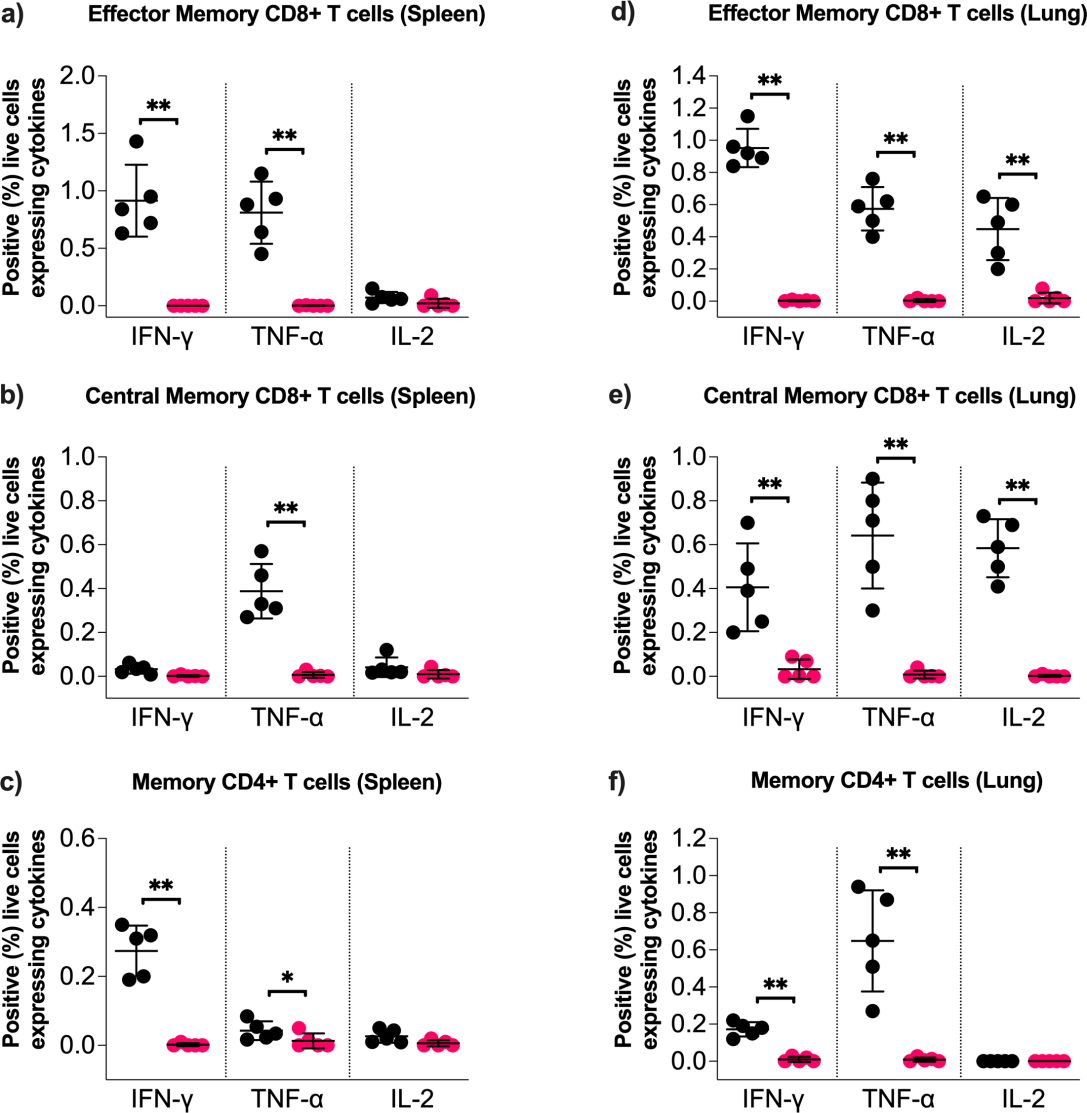
Memory T cell response induced by intranasal immunization. Female Balb/c mice were randomly divided into three experimental groups (*n* = 5) and immunized intranasally on days 0 and 21 with 1 × 10^9^ PFU of rAd5-SARS2-S1 or rAd5-GFP. Animals were euthanized on day 90. Splenocytes or lung cells were isolated and *ex vivo* re-stimulated with synthetic pooled peptides covering the ancestral Wuhan SARS-CoV-2 strain S1 protein for 6 h. Histograms show the percentages of IFN-γ^+^, TNF-α^+^, and IL-2^+^ effector CD8^+^CD44^hi^CD62L^−^ memory T cells (**a**) spleen and (**d**) lung, central CD8^+^CD44^hi^CD62L^+^ memory T cells in (**b**) spleen and (**e**) lung, and memory CD4^+^CD44^hi^CD62L^−^ T cells in (**c**) spleen and (**f**) lung. All the represented data were calculated by subtracting the values of the expressed levels by non-stimulated cells from the re-stimulated cells. Results are represented as mean ± SD. Statistical significance was determined by Mann–Whitney test and reported as *, *P* < 0.05; **, *P* < 0.01.

Memory T cells play a key role in limiting viral infections and protecting against disease. Thus, we examined systemic (spleen) and pulmonary (lung) memory CD8^+^ and CD4^+^ T cells in mice vaccinated with IN rAd5-SARS2-S1 or rAd5-GFP. Splenocytes and lung cells isolated from immunized mice on day 90 (10 weeks after boost) were re-stimulated *ex vivo* as indicated before, and the frequency of IFN-γ^+^, TNF-α^+^, and IL-2^+^ T cells was assessed using a similar gating strategy as shown in Fig. 3a. Similar to what have been observed with IM immunization, significant increase in IFN-γ^+^ and TNF-α^+^ TEM CD8^+^ T cells (Fig. 5a), TNF-α^+^ TCM CD8^+^ T cells (Fig. 5b), and IFN-γ^+^ and TNF-α^+^ memory CD4^+^ T cells (Fig. 5c) was seen in mice immunized with rAd5-SARS2-S1 compared to the rAd-GFP group. Importantly, rAd5-SARS2-S1 immunized mice showed markedly significant levels of pulmonary IFN-γ^+^, TNF-α^+^, and IL-2^+^ TEM (Fig. 5d) and TCM (Fig. 5e) CD8^+^ T cells as well as TNF-α^+^ memory CD4^+^ T cells (Fig. 5f). Thus, IN administration with two doses of rAd5-SARS2-S1 induces significantly high levels of systemic and local T cell responses in the spleens and lungs.

### Intramuscular immunization with the S1-based vaccine ameliorates disease severity in SARS-CoV-2 challenged Syrian hamsters

**Fig 6:**
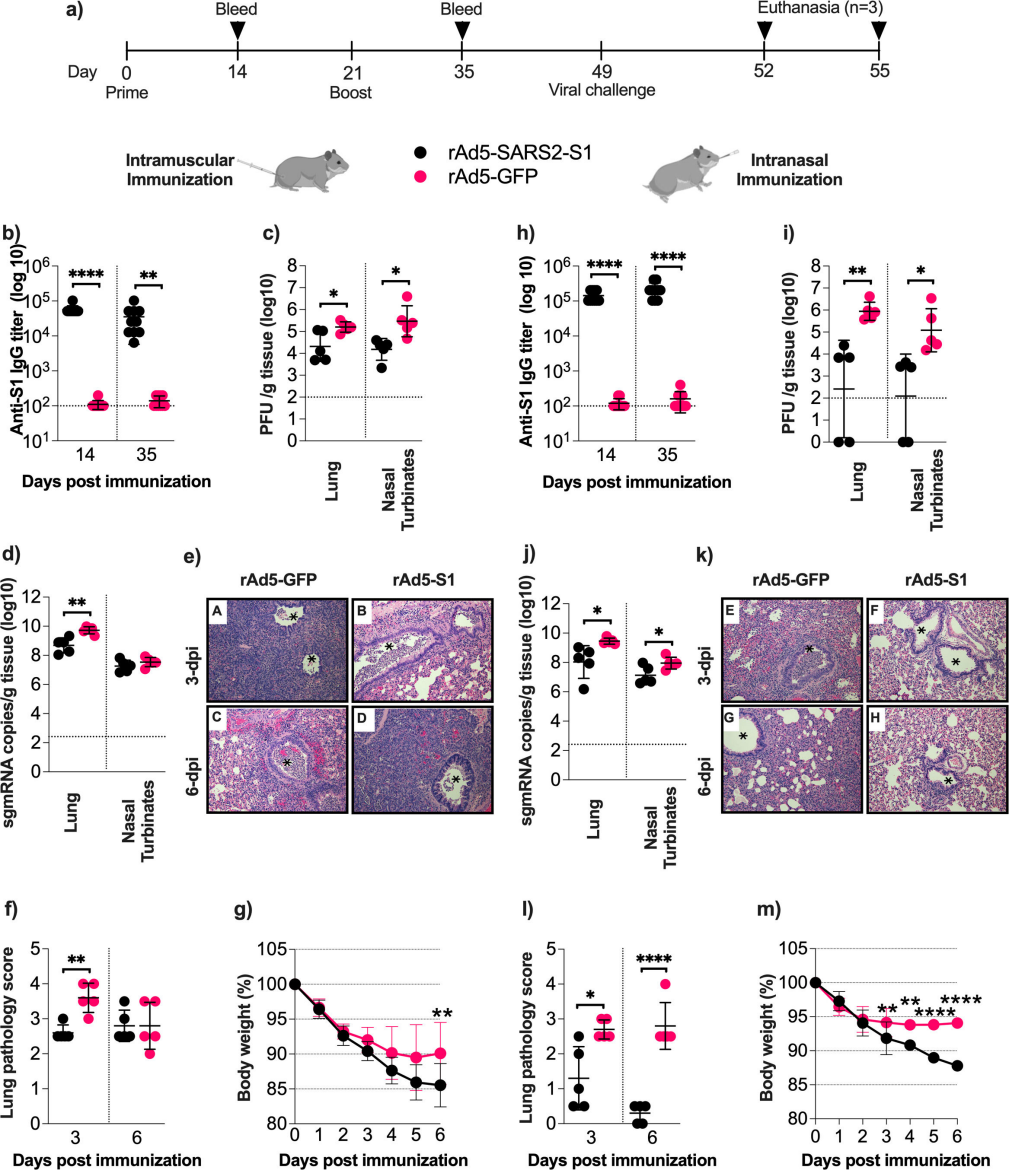
Protection of hamsters from viral replication and lung pathology. (**a**) Syrian hamsters were randomly divided into two experimental groups (*n* = 10) and immunized intramuscularly or intranasally on days 0 and 21 with 1 × 10^9^ PFU of rAd5-SARS2-S1 or rAd5-GFP. Blood samples were collected on days 14 and 35. Endpoint titers of S1-specific binding IgG in serum from hamsters immunized (**b**) intramuscularly or (**h**) intranasally were determined by ELISA. Animals were challenged intranasally with SARS-CoV-2 virus on day 49 and monitored for 6 days post-challenge. Hamsters were sacrificed 3 dpi (*n* = 5) and 6 dpi (*n* = 5), and their lungs and nasal turbinates were isolated for viral titration and evaluation of histopathological changes in the lungs. Infectious viral titers (pfu/gram of tissue) in lungs and nasal turbinates collected on day 3 were determined in each sample from hamsters immunized (**c**) intramuscularly or (**i**) intranasally using plaque assay, and titers were normalized per gram of tissue. Viral E sgmRNA copies (copies/gram of tissue) in lungs and nasal turbinates collected on day 3 were determined in each sample from hamsters immunized (**d**) intramuscularly or (**j**) intranasally via RT-qPCR, and titers were normalized per gram of tissue. Histopathological changes in the lung of (**e**) intramuscularly and (**k**) intranasally immunized hamsters challenged with SARS-CoV-2. * Bronchi or bronchioles. Histopathological scores of the lungs are shown from hamsters immunized (**f**) intramuscularly or (**l**) intranasally. Hamsters body weight was measured for 6 days following IN challenge and changes are shown compared to day 0 for (**m**) IM or (**k**) IN immunization. Statistical significance was determined by Mann–Whitney test for panels (**b, c, d, f, h, i, j and l**). Statistical significance was determined by two-way ANOVA analysis of variance for panels (**g and m**). Significance was reported as *, *P* < 0.05; **, *P* < 0.01; ****, *P* < 0.0001. Data are shown as mean  ±  SD; Dotted lines represent cutoffs of the assays.

To evaluate the protective efficacy of our vaccine, Syrian hamsters were vaccinated intramuscularly or intranasally (Fig. 6a) in a prime-boost vaccine regimen with 1 × 10^9^ PFU of rAd5-SARS2-S1 or rAd5-GFP. The vaccine elicited strong and significantly high humoral responses upon IM administration as expected based on our observations in mice (Fig. 6b). Next, animals were challenged 4 weeks after receiving their second immunization. rAd5-SARS2-S1 vaccination significantly reduced viral replication in the lungs as determined by infectious viral titer (Fig. 6c) and viral sgmRNA expression (Fig. 6d) 3 dpi when compared to the rAd5-GFP control. However, in the nasal turbinates 3 dpi, a significant reduction was only observed in infectious viral titer (Fig. 6c) and not in viral sgmRNA expression (Fig. 6d). Histopathologically (Fig. 6e), the lungs from hamsters immunized intramuscularly with rAd5-GFP (Photomicrograph A) showed moderate to severe acute bronchitis, bronchiolitis, and bronchopneumonia at 3 dpi. The lumens of many airways were occluded with large numbers of mixed neutrophils and mononuclear cells, and the alveoli in the surrounding areas collapsed. The lungs from hamsters immunized with rAd5-SARS2-S1 (Photomicrograph B) showed similar changes although the lung tissue was less consolidated at this time point. At 6 dpi, the lungs of intramuscularly immunized hamsters (Photomicrographs C-D), regardless of the vaccine, were similar in nature and consisted of subacute broncho-interstitial pneumonia and areas of alveolar collapse with very little aeration. These results were reflected in the lung pathology score as we found significantly lower pathology scores in the lungs 3 dpi but not 6 dpi (Fig. 6f). Furthermore, minor but significant recovery and weight gain were observed on 6 dpi in hamsters immunized with rAd5-SARS2-S1 in which they only lost 10% of their weight compared to 15% wt loss in control group 6 days post-challenge (Fig. 6g). These data suggest that IM vaccination rAd5-SARS2-S1 cannot afford full protection against SARS-CoV-2 challenge in hamsters.

### Intranasal rAd5-SARS2-S1 vaccine affords superior protection against SARS-CoV-2 challenge in Syrian hamsters

Next, we examined the protective efficacy of the IN route. We found higher S1-specific IgG antibodies compared to IM immunization (Fig. 6h). IN administration of rAd5-SARS2-S1 significantly reduced infectious viral titer (Fig. 6i) 3 dpi in the lungs and nasal turbinates compared to the rAd5-GFP vaccinated hamsters, in which it was lower than that observed with IM vaccine by 1.5–2 logs. IN administration also reduced the level of viral sgmRNA in both the lungs and nasal turbinates relative to the rAd5-GFP group (Fig. 6J) although the level of sgmRNA was comparable to the IM vaccinated group. Next, the right lungs were collected from the challenged hamsters 3 and 6 dpi and analyzed for histopathological changes (Fig. 6k). Changes in the lungs of hamsters intranasally immunized with rAd5-GFP 3 dpi (Photomicrograph E) or 6 dpi (Photomicrograph G) were similar in nature but with less severity compared to those of hamsters immunized with IM rAd5-GFP 3 dpi (Photomicrograph A) or 6 dpi (Photomicrograph C), respectively. They showed an overall high pathological score reflective of severe histopathological changes on both time points (Fig. 6l). Conversely, IN rAd5-SARS2-S1 vaccine reduced lung pathology significantly 3 dpi which further improved significantly on 6 dpi compared to rAd5-GFP group (Fig. 6l). Specifically, the lungs from rAd5-SARS2-S1 groups 3 dpi (Photomicrographs F) showed only mild acute bronchopneumonia and focal alveolar collapse (Fig. 6k). This was followed by an almost complete resolution of pulmonary inflammation although focal collapse of alveolar spaces was observed in some areas (Photomicrographs H). Animal weight was measured daily post-challenge, with all groups experiencing an initial weight loss after challenge in which rAd5-GFP control group continued to rapidly lose weight reaching ~12% wt loss by day 6 post-challenge (Fig. 6m). Importantly, the IN rAd5-SARS2-S1 vaccine resulted in significantly faster weight gain starting 3 days post-challenge relative to the control group, with the mean weight of SARS2-S1 immunized hamsters reaching ~94% 6 dpi (Fig. 6m). Together, these data suggest that mucosal delivery of rAd5-SARS2-S1 affords better protection than intramuscular administration of the same vaccine.

## DISCUSSION

The COVID-19 pandemic has made vaccine development an international priority. Several efficacious SARS-CoV-2 vaccines have been developed and pre-qualified by the WHO and authorized by various health jurisdictions (1–5). Most approved SARS-CoV-2 vaccines or those under development are based on IM administration. These vaccines elicit high levels of circulating antibodies, memory B cells, and CD4^+^ and CD8^+^ T cells but are inefficient in inducing mucosal immunity (14, 30, 31). In addition to driving strong systemic response, IN vaccines such as rAd-based SARS-CoV-2 vaccines can induce potent mucosal immune responses at the sites of infection, decreasing transmission and reducing disease severity (5, 17, 20, 26–31). Such IN vaccines could be used as boosters to current or future vaccines or used in combined heterologous immunization regimens with IM vaccines to further enhance immunity and protection (26, 27, 47).

Here, we preclinically explored IM and IN delivery of our SARS-CoV-2 vaccine candidate rAd5-SARS2-S1 in mice. We demonstrated that the vaccine could elicit long-lasting and does-dependent serum antibody response upon IM vaccination. This response was characterized by Th1-polarization, as demonstrated by significantly high levels of antigen-specific IgG2a:IgG1 and IgG2b:IgG1 ratios and dominated by RBD-specific antibodies. Additionally, the vaccine elicited nAbs against several SARS-CoV-2 variants. Although significant decline in levels was observed over time, high nAbs titers were maintained until day 90 (i.e., 10 weeks post-boost). IM vaccination also resulted in the induction of high levels of durable systemic cytokine-producing effector and central memory CD8^+^ T cells and memory CD4^+^ T cells that lasted up to 10 weeks after boosting. Similarly, IN vaccination with rAd5-SARS2-S1 also elicited significant levels of systemic Th1-biased S1-binding IgG, nAbs, and memory T cells similar to IM vaccination. Moreover, it induced mucosal humoral and cellular responses in the BALF and lungs, respectively. Interestingly, the IN vaccine elicited less pronounced IgG2a:IgG1 and IgG2b:IgG1 ratios compared to the IM vaccine, primarily because of higher IgG1 levels induced following IN administration. Furthermore, the nAb titers were lower by ~50% in the intranasally vaccinated animals compared to the IM group.

Furthermore, we evaluated the protective efficacy of rAd5-SARS2-S1 in Syrian hamsters. The rAd5-SARS2-S1 vaccine elicited significantly strong humoral immune responses upon both IM and IN administration relative to vector controls after two doses. Despite being immunogenic and reducing viral burden in the lungs (infectious particles and viral RNA) and nasal turbinates (infectious particles), IM vaccination with rAd5-SARS2-S1 only slightly ameliorated disease severity, as indicated by reduced lung pathology at 3 dpi and slight recovery from weight loss by day 6.

IN administration of rAd5-SARS2-S1 significantly reduced both infectious particles and viral RNA in both the lungs and nasal turbinates 3 dpi. While the levels of sgmRNA were similar to those observed with IM vaccination, a more pronounced effect in controlling viral replication in the nasal turbinates was observed. IN administration also reduced lung pathology significantly 3 dpi and nearly resolved histopathological changes and pulmonary inflammation 6 dpi. Importantly, animals receiving rAd5-SARS2-S1 intranasally showed faster and more significant recovery from weight loss starting from day 3 post-challenge compared to both the control GFP group and IM vaccinated group. These results suggest that IN delivery could be a superior approach for vaccination as it induces both systemic and local humoral immunity thereby reducing viral replication in both the upper and lower respiratory tract.

Several studies have evaluated mucosal COVID-19 vaccines preclinically, demonstrating the encouraging immunogenicity and protection of the approach (17, 18, 20, 23, 24, 26–31). Most of these studies have utilized adenovirus-based platforms. While clinical trials of some of these vaccines had inconsistent results (19–22), two IN adenovirus-based vaccines were licensed in China and India (5, 26, 27). A recent systematic review and meta-analysis of clinically tested IN COVID-19 vaccines highlighted the favorable safety profile of IN vaccination although inconsistent immune responses and protective efficacy were observed after primary vaccination (25). However, use of IN vaccines as a booster dose showed more promising results in which levels of nAbs and mucosal immunity were higher than those observed following IM vaccination (25). Nonetheless, clinical data on IN vaccines are scarce and more studies are clearly needed to further study long-term safety and protective efficacy.

It is hypothesized that using the S1 subunit of the coronavirus S protein as a vaccine can focus the immune response on the neutralizing epitope-rich regions such as the RBD and NTD to elicit protective immunity while eliminating the risk of ADE due to non-neutralizing antibodies (34, 35, 48). Several S1-based vaccines for MERS-CoV (33, 49) and SARS-CoV-2 (43, 50–54) have been developed using different platforms and animal models. Compared to MERS-CoV S1 vaccines which showed potent immunogenicity and protective efficacy, results of SARS-CoV-2 S1-based vaccines have varied greatly between studies. While some studies reported low-to-medium immunogenicity (43, 51) or failed to provide protection (53, 54), others induced high levels of nAbs and protected animals (50, 52). Our data, along with many of these previous reports, clearly demonstrate the potential of SARS-CoV-2 S1 as a promising immunogen.

Pre-existing immunity to adenoviral vectors is an important consideration, particularly in populations with high rates of natural adenovirus exposure and in the context of repeated dosing or booster strategies as the pre-existing anti-adenovirus antibodies may reduce the efficacy of rAd5-vectored vaccines. Pre-existing human Ad5 and chimpanzee adenovirus antibodies were reported at high rates in several parts of the world (55, 56). Clinical data also suggest that high (>1:200) but not low titers of Ad5 nAbs could interfere with seroconversion, lower immune response levels, and shorten the vaccine persistence (57). Nonetheless, high dose of IM rAd5-Ebola vaccine was shown to be sufficient to elicit nAbs in adenovirus individuals seropositive for adenovirus (58, 59). Here, we found significantly higher humoral response levels upon boosting in animals despite the administration route (IM or IN), vaccine dose (Fig. 1), and the presence of anti-rAd5 antibodies in primed and boosted animals (data not shown). Additionally, viral vectors, such as rAd5, can be delivered through heterologous combinations of prime-boost regimens using distinct adenovirus serotypes or different routes including mucosal delivery, which has been reported to be less affected by pre-existing immunity (60–63). Indeed, our data support this observation, as we found higher levels of S1-specific IgG by at least 1–2 logs when comparing IN versus IM immunization. Thus, the possible impediment of seroconversion by pre-existing anti-adenovirus immunity could be mitigated by mucosal delivery or heterologous prime-boost regimens with viral vectors of nonhuman-origin or less common serotypes.

While we tested our vaccine candidate *in vivo* only against the wild-type strain of SARS-CoV-2, we demonstrated that it could elicit circulating and mucosal nAbs against several variants. Thus, future studies examining this vaccine or other modified versions using updated sequences from emerging variants, more dominant circulating strains, and/or conserved target antigens are warranted. Additionally, further studies are needed to assess the efficacy of this vaccine using animal models of different ages and immune statuses. It is of note that while we investigated long-term immunogenicity in the murine model (~10 weeks after boost), we only assessed immunogenicity and protection efficacy over a short duration post-vaccination in hamsters. Thus, further studies are clearly needed to evaluate the long-term durability of the vaccine’s protective effects, particularly when using the IN route. We observed potent mucosal IgA responses in the intranasally immunized group. While we do not expect to find marked induction of mucosal IgA levels in the IM group, they should be tested in future studies to provide a more complete comparison between IN and IM vaccines.

In conclusion, this study demonstrates that the rAd5-SARS2-S1 vaccine is immunogenic in mice and hamsters when administered intramuscularly or intranasally. While IM vaccination can ameliorate disease severity in animals, IN administration induces potent mucosal and systemic immunity and provides superior protection . The efficient induction of systemic and mucosal immunity by this vaccine holds promise in efforts to combat against potential outbreaks or emerging pathogens.

## Data Availability

All relevant data are within the paper and are available upon request from the corresponding authors.
